# The Mosquito, Its Birth, Life and Death

**Published:** 1870-09

**Authors:** 


### THE MOSQUITO, ITS BIRTH, LIFE AND DEATH.

If stagnant water is exposed to a summer's sun for several days, waggletails begin to appear on the surface and grow until near a quarter of an inch long, seeming to live on air and water; in a short time they become encased and sink to the bottom; in a few hours a short hair grows out of the sides, soon becoming a small caterpillar, rising to the surface and floating to the shore by the aid of the slightest breath of air. Soon a fly is hatched, leaving its tiny shell on the water; that fly is the mosquito, which is ready to suck the very life-blood out of you. Where mosquitoes abound, there is but one certain and efficient means of keeping them from disturbing sleep, — get under a good mosquito-bar, which should be adjusted to the bed before sundown, for they do not begin to stir around in rooms until later in the evening.

If there are but two or three mosquitoes in the room, or within your bar, the cheapest and least troublesome way of getting rid of them is to let them alone when they alight on you, until they have inserted their sting clear down into the skin, known by the sensation calling for a scratch, move the hand very slowly to the spot, and when within four or five inches of your enemy, crush it with a rapid movement. The insect then seems so intent on drinking the blood that it is unwilling to forego the pleasure, or the sting is so far inserted that it cannot be withdrawn in an instant. If this method is not pursued, two or three mosquitoes will keep one awake half the night, and as mad as a hornet besides, with the result that the little fellow gets his supper at last, for he worries you until you fall to sleep from mere exhaustion, and then he feeds upon you at his leisure, and when he is full, withdraws his lance, flies to the top of the bed or canopy, and laughs you to scorn, or will rest with complacency until he dies or gets hungry again ; it is said, with what truth we know not, that with one fill of blood he feeds no more ; it is the triumph of his life.

Mosquitoes must have a living as well as other people, and what is more, they will have it, and will go through fire and water to get it. We once noticed a device at Key West to keep them away ; a woman sitting out of doors near a pile of burning rags, the wind blowing the smoke on her ; she could stand that stench by the hour, but the mosquitoes wouldn't, they had more sense.

